# Differentially Expressed Extracellular Vesicle, Exosome and Non-Exosome miRNA Profile in High and Low Tick-Resistant Beef Cattle

**DOI:** 10.3389/fcimb.2021.780424

**Published:** 2021-12-17

**Authors:** Pevindu Abeysinghe, Natalie Turner, Hassendrini Peiris, Kanchan Vaswani, Nick Cameron, Nathanael McGhee, Jayden Logan, Murray D. Mitchell

**Affiliations:** ^1^ Centre for Children’s Health Research, School of Biomedical Sciences, Faculty of Health, The Queensland University of Technology, Brisbane, QLD, Australia; ^2^ Nindooinbah Pastoral Company, Nindooinbah, QLD, Australia

**Keywords:** extracellular vesicles, exosome, tick, beef cattle, miRNA

## Abstract

Heavy tick burden on beef cattle account for huge economic losses globally, with an estimated value of US$22-30 billion per annum. In Australia, ticks cost the northern beef industry approximately A$170-200 million. Methods to evaluate and predict tick resistance would therefore be of great value to the global cattle trade. Exosomes (EX) are small extracellular vesicles (EVs) of ~30-150nm diameter and have gained popularity for their diagnostic and prognostic potential. EX contain, among other biomolecules, various types of RNA including micro-RNA (miRNA) and long noncoding RNA (lncRNA). MiRNA specifically have been validated as therapeutic biomarkers as they perform regulatory functions at the post-transcriptional level and are differentially expressed between divergent groups. The objective of the present study was to evaluate the miRNA profiles of EV and fractionated exosomal samples of high and low tick-resistant beef cattle to highlight potential miRNA biomarkers of tick resistance. Cows (*n* = 3/group) were classified into high or low tick resistant groups according to a novel scoring system. EVs and EX were isolated and fractionated from the blood plasma of high and low tick resistant cattle using established isolation and enrichment protocols. The resultant EX and non-EX samples were processed for next generation miRNA sequencing. Offspring of the cows in each high and low tick resistant group underwent the same processing for blood plasma EX, non-EX and miRNA analysis to evaluate the heritability of miRNA associated with tick resistance. A total of 2631 miRNAs were identified in EX and non-EX fractionated samples from high and low tick-resistant beef cattle. MiR-449a was highly expressed in maternal high tick-resistant EX samples. Of these, 174 were novel miRNAs, and 10 were differentially expressed (DE) (FDR < 0.05). These 10 DE miRNAs were also present in EVs, and three miRNAs were highly expressed: miR-2419-3p, miR-7861-3p and miR-2372-5p. Although 196 novel miRNAs were identified in fractionated samples of offspring, no miRNA were differentially expressed in these animals.

## 1 Introduction

Ticks pose a considerable threat to livestock globally, specifically in beef cattle farming. It has been estimated that 80% of the world’s cattle are at risk, with US$20 to US$30 billion economic losses per annum link to tick and tick-borne diseases (Lew-Tabor and Rodriguez Valle, 2016; Burrow et al., 2019). In Australia, the tick burden costs the northern beef industry around A$170-200 million (Playford et al., 2005). Tick infestation causes stress and weakens the immune system, which affects the performance of the beef cow (Mapholi et al., 2014). Each engorging female tick is responsible for an average 1.37 ± 0.25 g bodyweight loss in *Bos taurus* cattle (Jonsson, 2006). Tick-borne diseases increase cattle mortality, chronic morbidity, and treatment costs (Jonsson et al., 2008; Hurtado and Giraldo-Ríos, 2018). Farm management systems use conventional options such as acaricides to control tick infestation, however this is not a sustainable strategy in the long term (Mapholi et al., 2014). Intensive usage of acaricides causes pressure on pasture systems and leads to selection for acaricide-resistant tick populations (Kasaija et al., 2021). Lack of understanding on the whole genome of parasites and antigenic variation challenge sustainable use of parasite vaccines against ticks (Barré et al., 2011; Lew-Tabor and Rodriguez Valle, 2016). Farmers utilize different grazing management techniques such as pasture rotation and pasture burning to reduce exposure and control tick populations (Kasaija et al., 2021). Unfortunately, climactic conditions desirable for cattle herds are also ideal for tick propagation, and grazing herds are more susceptible to heavy tick burden (Young et al., 1988; Kasaija et al., 2021). Cattle-ticks represent a top priority endemic disease for the red meat industry in Australia (Lane et al., 2015).

Tick burden affects not only cattle but is also linked to human diseases. For instance, a glycoprotein from tick saliva, α-Gal, causes an allergic condition termed α-Gal syndrome in humans, resulting in delayed hypersensitivity to consumed red meat products (Cabezas-Cruz et al., 2019). Interestingly, some cattle exhibit a natural resistance to ticks and carry a low tick burden (Burrow et al., 2019). The study of the physiological or genetic mechanisms that confer this natural resistance offers an opportunity to identify alternative and more effective tick control methodologies (Marima et al., 2020). The level of tick resistance varies among different cow breeds (Marufu et al., 2011; Marima et al., 2020). The cattle tick resistance is considered as a polygenic trait which includes morphological, physiological and behavioural traits, and, therefore heritability plays a main role (Mapholi et al., 2014). These factors suggest genetic selection is a considerable option towards the development of a sustainable cattle tick control methodology.

Extracellular vesicles (EV) are a heterogenous group of nanoparticles that originate from the endosomal sorting complex required for transport (ESCRT) pathway, or shed directly from the plasma membrane (van Niel et al., 2018). EVs are classified into subpopulations by their origin, size, morphology, and protein markers specific to each subtype. Exosomes (EX) are an EV subtype of diameter ~30 – 150 nm and carry unique molecular cargo that has been used in biomarker development and targeted therapeutics (Zhang et al., 2019; Abeysinghe et al., 2020; Dai et al., 2020; Kugeratski et al., 2021). Exosomal cargo contains complex functional molecules ranging from proteins (Crookenden et al., 2016), lipids (Record et al., 2014), mRNAs (Valadi et al., 2007) and miRNAs (Valadi et al., 2007). Differential expression of exosomal miRNA have been the focus of numerous studies involving divergent groups (Colitti et al., 2019; Ma et al., 2019; Wang et al., 2019). EX are intercellular communicators (Zhang et al., 2019) and are associated with major cellular processes like signal transduction (Gangoda et al., 2015), immune responses (Greening et al., 2015) and antigen presentation (Mittelbrunn et al., 2011). EX can be transferred from mother to fetus *via* the placenta, which supports the idea that EX are important for maternal-fetal communication (Sheller-Miller et al., 2016).

While a previous study identified single nucleotide polymorphisms and chromosome segments associated with tick burden, to date there have been no biomarkers or genetic variants identified to account for tick resistance (Porto Neto et al., 2011).

In this study, high and low tick resistant beef cattle were classified according to a novel tick scoring system. Next-generation miRNA sequencing was carried out on plasma-derived EX and non-EX particles from high and low tick cattle to evaluate their miRNA profiles and assess differential expression of miRNA.

## 2 Materials and Methods

### 2.1 Animals, Management and Blood Collection

The animals, management, and sample collections were approved by the Animal Welfare Unit, UQ Research and Innovation, the University of Queensland (UQCCR/459/16). A total of 199 animals were selected randomly and tick scores were given accordingly (**Table 1**).

**Table 1:** Final tick scores of cow population.

Final score * Week ended cross tabulation									
Count									
		Week ended						Total	Score % total
		1	2	3	4	5	6		
Final score	1			1	0	1	91	93	44.2857
	2	0	0	2	6	0	33	41	19.5238
	3	0	14	3	18	0	3	38	18.0952
	4	7	1	2	7	0	2	19	9.04762
	5	1	0	0	7	0	0	8	3.80952
Total		8	15	8	38	1	129	199	

**Figure 1:**
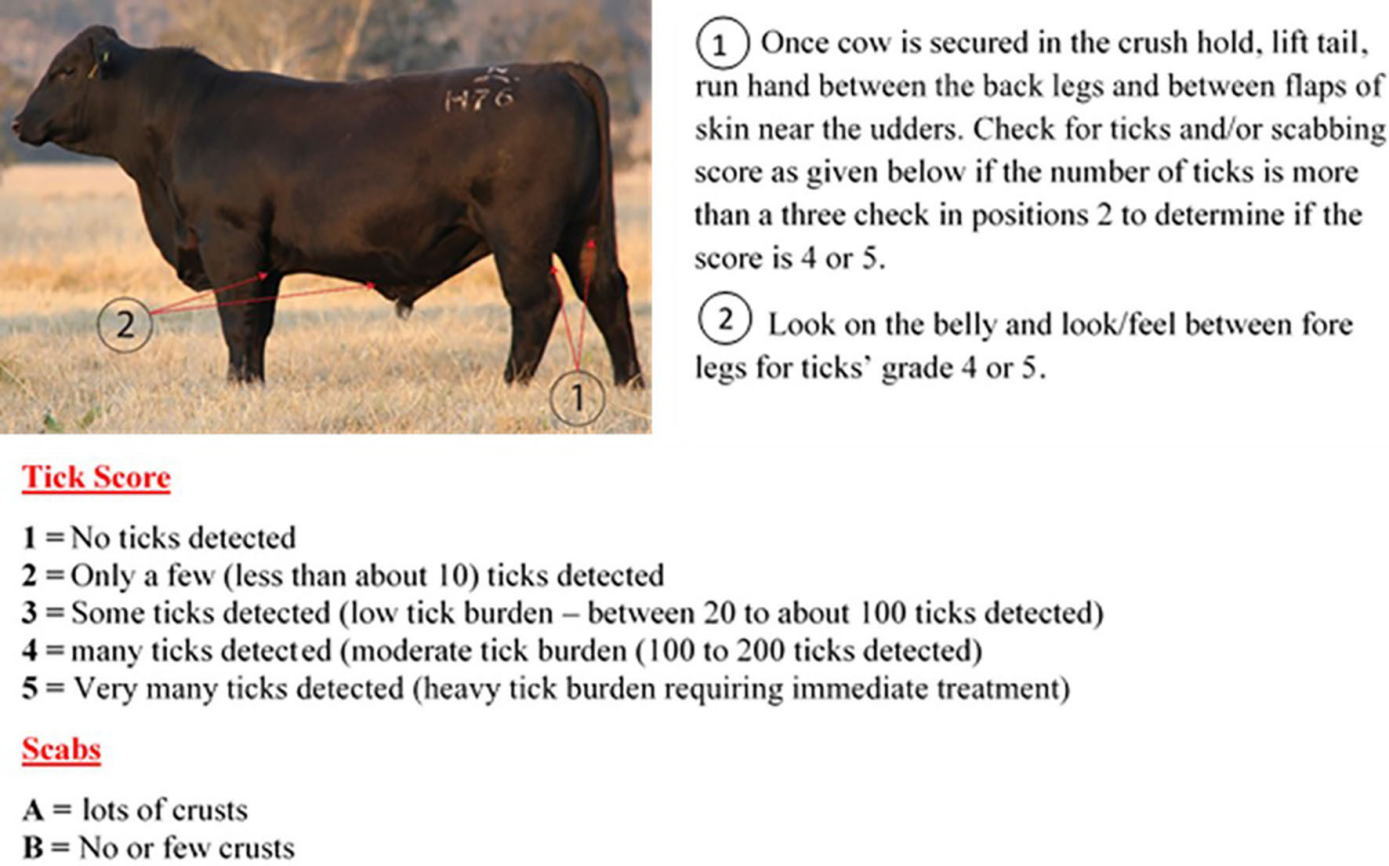
Cow tick scoring methodology based on the number of ticks and scabs detected in the body of the cow.

The animals were carefully examined for evidence of tick infestation as part of a thorough physical exam. Animals were hand checked for the presence and absence of ticks on their hind regions and belly over a three-month period. A scoring system was developed (1-5, A or B), (1) no identifiable tick burden, (2) < 10 ticks, (3) 20 to 100 ticks, (4) 100 to 200 ticks, (5) > 200 ticks with (A) representing crusting and (B) no crusting (**Figure 1**). Animals with a score of >3 were treated as part of the commercial program and those with <3 untreated.

Blood was collected from mother/sire and offspring in EDTA vacutainer tubes and plasma separated by centrifugation at 3000 x *rcf* for 10 min at 4°C. Plasma was stored at -80°C until required for EV/EX isolation. Detailed information of sire and dam histories and other relevant information (e.g. last tick treatment, weight, pasture location) was recorded as part of the commercial program.

From this larger animal group, 3 high tick-resistant (1B) and 3 low tick-resistant (5A) mother/sire and offspring (highest and lowest tick burden animals) blood plasma were used for EV and EX isolation.

### 2.2 Extracellular Vesicle (EV) Isolation

EVs were isolated from the blood plasma of animals using as established sequential centrifugation protocol (Koh et al., 2018). Briefly, plasma was centrifuged at 2,000 x *rcf* for 30 min at 4°C and 12,000 x *rcf* for 30 min at 4°C to remove cellular debris and apoptotic bodies. It was then filtered through a 0.22-μm polyether sulfone membrane filter (Corning Inc., Corning, NY) and then ultracentrifuged at 100,000 x *rcf* for 2 hr at 4°C. Finally, the pellets containing the EVs were resuspended in 500 μl Dulbecco’s Phosphate Buffered Saline (DPBS, pH 7.0 – 7.2; Gibco, Life Technologies Australia Pty Ltd) and stored at -80°C for further analysis.

### 2.3 EX Isolation and Characterization

#### 2.3.1 Size Exclusion Chromatography (SEC)

EX were isolated from plasma by ultracentrifugation and size exclusion chromatography (SEC) as previously described (Koh et al., 2018). The high and low tick resistant samples from mother/sire and offspring were pooled separately. Briefly, 500 µL EV suspensions resulting from sequential centrifugation were fractionated using qEV original size exclusion columns (Izon Science, New Zealand). Individual 500 µL fractions were eluted from the column and collected in separate 1.5 mL microcentrifuge tubes (a total of 16 fractions), as per manufacturer’s instructions. The fractions were collected as follow s; 1 – 6 as void volume and particles >200 nm, 7 – 10 as exosomal (EX) fractions (particles <200 nm), and 11 – 16 as soluble proteins (non-EX) fractions. One column was used per animal groups to maintain group heterogeneity. In between uses, the columns were flushed with 0.5 mL 1M NaOH solution, followed by 15-20 mL filtered DPBS.

Three EX and non-EX samples from each of extreme high (1b) and low (5a) tick resistant groups were pooled for SEC miRNA analysis.

Quantification of protein concentration of SEC fractions was evaluated using Bicinchoninic Acid (BCA) assay (Sigma-Aldrich, St Louis, MO, USA) and bovine serum albumin (Sigma-Aldrich, St Louis, MO, USA) dilutions were used as standards. The size distribution by Nanoparticle tracking analysis was conducted as previously described (Koh et al., 2018).

### 2.4 Exosomal miRNA Isolation

EX fraction 7-10 (EX) and 11-16 (Non-EX) pooled and samples were incubated with TRIzol at room temperature for 5 minutes (2.5 volume of sample: 7.5 volume of TRIzol). Chloroform was added (2.5 volume of sample: 1.5 volume of Chloroform) and centrifuged for 15 min at 4°C, at 12,000 *rcf*. The upper aqueous layer was carefully transferred to a new microcentrifuge tube and 1.5 volumes of 100% ethanol was added. This mixture was passed through a miRNeasy mini column (miRNeasy mini kit, 217004, QIAGEN) and miRNA was isolated according to the manufacturer’s protocol.

### 2.5 MiRNA

#### 2.5.1 Sequencing and Data Analysis

Isolated miRNA samples were sent to the Australian Genome Research Facility (AGRF) for next generation sequencing. NEBNext^®^ Multiplex Small RNA Library Prep Set for Illumina^®^ was used for library preparation and Novaseq S1 platform was used for single end 100bp sequencing. A quality control was measured for each sample and samples greater than 78.19% bases above Q30 were selected. The reads were also screened for the presence of any Illumina adapter/overrepresented sequences (adapter sequence as per the library preparation kit: AGATCGGAAGAGCACACGTCTGAACTCCAGTCAC) and cross-species contamination. Reads were trimmed and length filtered using Trim Galore! to be between 14 and 38 base pairs long. The cleaned sequence reads were then aligned against the *Bos taurus* genome (Build version UMD3.1). The STAR aligner (v2.5.3a) was used to map reads to the genomic sequences (https://github.com/alexdobin/STAR/blob/master/doc/STARmanual.pdf) and alignment files were in BAM format. The counts of reads mapping to each known miRNA were identified using unitas (https://sourceforge.net/projects/unitas/). The differential gene expression was performed using edgeR (version 3.30.3) of R package 4.0.3 (https://bioconductor.org/packages/release/bioc/html/edgeR.html). False discovery rate (FDR) analysis was performed to correct for multiple hypothesis testing and set to 0.05 (FDR<0.05). Only miRNA meeting the FDR cut-off were considered statistically significant. Further, miRNAs meeting the FDR cut-off were filtered with log fold change (logFC), in which the upregulated DE miRNAs were considered if the logFC ≥ 1.5 and logFC ≤ −1.5 for downregulated DE miRNAs.

For volcano plots, cut offs were -log10(FDR) ≤ 1.3 and fold change threshold values were −1.5 ≤logFC ≥ 1.5.

MiRDeep2 was used to identify novel miRNAs in each sample https://www.mdc-berlin.de/n-rajewsky#t-data,software&resources (Friedländer et al., 2012). (Reference genome: UMD3.1; Hairpin: Bos taurus (Btau_5.0.1) hairpin obtained miRbase; Mature: Bos taurus (Btau_5.0.1) mature miRNA obtained from miRbase; Other mature: mature miRNA from related organisms, i.e. *Capra hircus* (CHIR_2.0) and *Ovis aries* (Oar_v4.0); UCSC browser species: Cow).

#### 2.5.2 Pathway Analysis

Four different miRNA target prediction tools (1. miRanda from miRNet version 2.0 (https://www.mirnet.ca/) (Fan et al., 2016); 2. miRmap (http://mirmap.ezlab.org.) (Vejnar et al., 2013); 3. TargetScanHuman 8.0 (http://www.targetscan.org/vert_80/) (Agarwal et al., 2015); 4. miRWalk (http://mirwalk.umm.uni-heidelberg.de/search_mirnas/) were used to identify multiple target binding sites of the 10 differentially expressed Mother SEC (EX and non-EX) miRNAs.

In miRNet target prediction, the Bos taurus was used as organism, miRBase ID as ID type and target type was miRanda genes. For Degree filter “All network nodes” were selected with a cut off value of greater than 3, greater than 100 cutoff was used for betweenness, and all networks were to connect with the shortest path (Herrnreiter et al., 2021).

Bos taurus was used as the organism for miRmap and 10 DE miRNAs were checked for targets individually. Targets which were above 95% of power exact was chosen as confident miRNA targets. All the individual miRNA targets were pooled together to make a total list of miRmap targets of 10 DE miRNAs (Vejnar et al., 2013).

In TargetScanHuman 8.0, individual miRNAs were used as inputs and only broadly conserved sites were identified as confident miRNAs (poorly conserved sites and other miRBased annotations were not included due to potential false positive targets). Next the gene target results were lifted from Human GRCh38/hg38 to ARS-UCD 1.2 bosTau9 genome using the UCSC table browser – Lift Genome Annotation feature (https://genome.ucsc.edu/cgi-bin/hgLiftOver). For this > 0.1 ratio of conserved bases used as the minimum ratio (Scheel et al., 2017). Next, the individual miRNA targets were pooled together to make a total list of miRNA targets.

MiRWalk, putative target genes of 10 DE miRNAs were predicted with a cutoff binding probability > 0.98 (Li et al., 2021).

Intersection of target genes from four miRNA target prediction tools were used as the confident target sites of differentially expressed miRNAs from Mother SEC samples (EX and non-EX samples). Genes shared with at least 2 target prediction tools were selected as the confident target genes.

The search tool for retrieval of interacting genes (STRING) (https://string-db.org) database was utilized for generation of protein-protein interaction (PPI) network for the intersected target genes (Szklarczyk et al., 2019). Using the STRING database, genes with a score ≥ 0.4 (Karimizadeh et al., 2019; Li et al., 2020) and species “*Bos taurus*” were chosen to build a network model visualized by Cytoscape (v3.9.0) (Shannon et al., 2003). Protein subcellular localization was visualized using compartments feature in StringDB (Binder et al., 2014).

## 3 Results

### 3.1 Nanoparticle Tracking Analysis (NTA)

**Figure 2:**
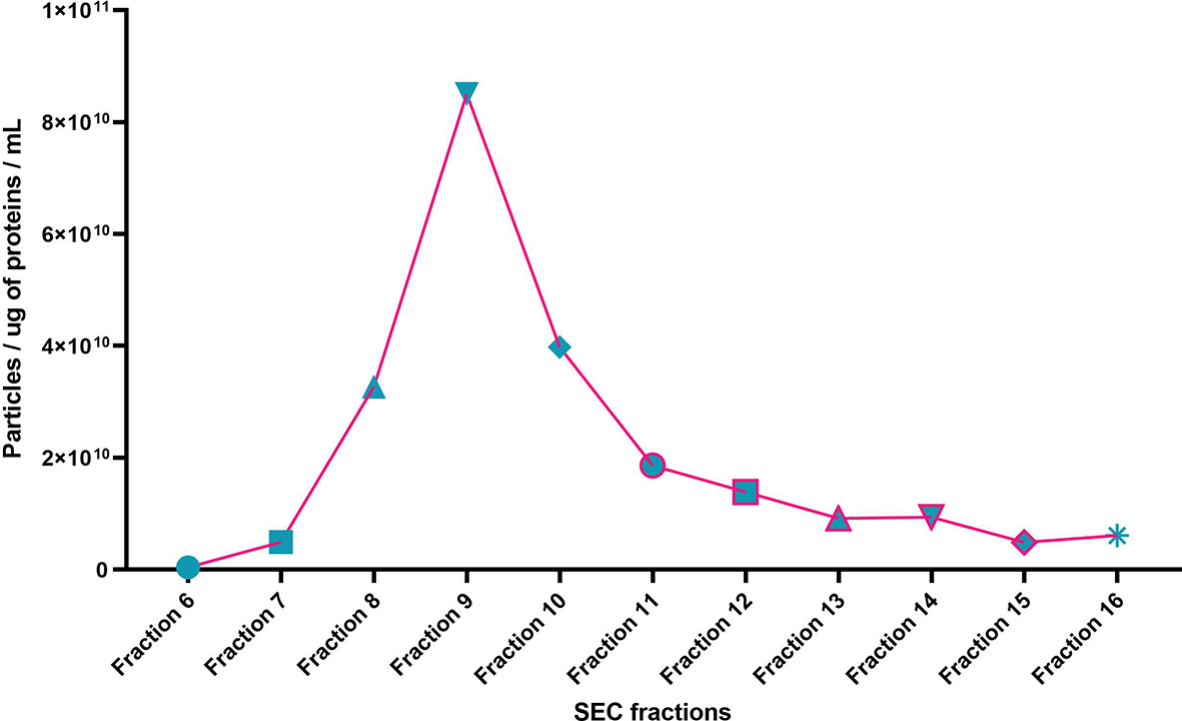
Particle size distribution per total protein for SEC fractions 6 – 16 of a representative sample (NTA particles per mL / total protein (µg/mL). Particle per ug of protein per mL.

Individual EX and non-EX fractions were assessed for their particle concentration (**Figure 2**). EX fractions 7 – 10 show the highest particle concentrations in fractions 8 – 10 (~4 x 10^10^ – 8 x 10^10^ particles/mL). Non-EX fractions 11 – 16 generally had a particle concentration < 2 x 10^10^ particles/mL.

### 3.2 Quality Assessment for Exosomal miRNA Samples From High and Low Tick Resistant Beef Cow Blood Plasma

All the samples yielded more than 100pg (0.1 ng) of miRNA according to quality control testing (**Table 2**).

**Table 2:** Bioanalyzer quality control (QC) results for miRNA samples.

Sample Type				miRNA Concentration [pg/μl]	miRNA/Small RNA Ratio [%]	miRNA yield [ng]
ID	Animal type	Vesicle type	Tick resistance			
A1	Mother	EV	Low	2402.7	67	189.81
A2	Mother	EV	High	446	17	35.23
A3	Mother	EV	Low	296.4	26	23.42
A4	Mother	EV	High	508.9	20	40.20
A5	Mother	EV	Low	211	20	16.67
A6	Mother	EV	High	119.4	11	9.43
A7	Offspring Master pool	EX	High	18.9	20	1.49
A8	Offspring Master pool	non-EX	High	50.3	14	3.97
A9	Offspring Master pool	EX	Low	2.3	3	0.18
A10	Offspring Master pool	non-EX	Low	35.5	15	2.80
A11	Mother Master pool	EX	High	66.7	8	5.27
A12	Mother Master pool	non-EX	High	5657.4	48	446.93
A13	Mother Master pool	EX	Low	86.1	7	6.80
A14	Mother Master pool	non-EX	Low	88.5	18	6.99

### 3.3 miRNA Expression Profiles

#### 3.3.1 SEC (EX and Non-EX) miRNA Profiles- Mother/Sire – High vs. Low Tick Resistant

**Figure 3:**
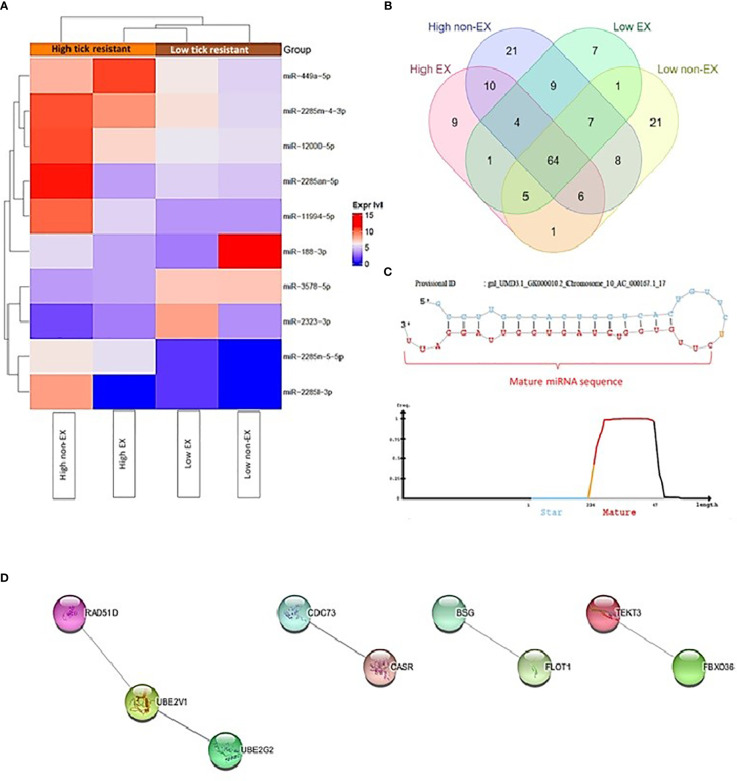
**(A)** Heatmap of differential expression of miRNAs between high and low tick resistant mother plasma SEC samples (EX and non-EX) **(B)** Novel miRNAs distributed among high and low tick resistant mother plasma SEC samples (EX and non-EX) **(C)** An exemplary novel miRNA loop which shows the predicted mature miRNA sequence **(D)** Protein-Protein interaction (PPI) networks generated by StringDB for the 37 gene targets predicted by miRmapper, TargetScan, miRmapper & miRWalk (Singletons are not included on the figure).

2632 miRNAs were identified in high and low tick resistant cattle. 2458 miRNAs were identified in miRbase database, and 174 novel miRNAs were identified by miRDeep2. The total list of miRNAs including the novel miRNAs are attached in **Supplementary file 1** (Abeysinghe, 2021). **Figure 3A** shows DE miRNA between high and low tick-resistant cattle, of which there were 10 DE miRNA in total. Specifically, mir-449a-5p, miR-2285-4-3p, miR-12000-5p were more highly expressed in high tick-resistant cattle, and miR-3578-5p, miR-2323-3p were more highly expressed in low tick resistant cattle (EX and non-EX fraction). In non-EX samples, low tick-resistant show a higher expression level of miR-188-3p and miR-3578-5p compared to high tick resistant. Most of the novel miRNAs were common to both groups, however 9 and 7 novel miRNAs were unique to high-tick resistant and low-tick resistant EX samples, respectively (**Figure 3B**). The sequence fragment with the highest read counts from the precursor miRNA loop was considered as the novel mature miRNA (**Figure 3C**). The total list of gene targets predicted by miRWalk, miRanda, TargetScan and miRmap are shown in **Supplementary file 2** (Abeysinghe, 2021). There were 37 shared genes which are shared with at least 2 target prediction [**Supplementary file 3** (Abeysinghe, 2021)]. Out of those 37 target genes, 16 genes were appeared in protein subcellular localization visualized by compartment analysis of stringDB (**Table 3**) and the protein-protein interaction (PPI) networks are shown in **Figure 3D**.

**Table 3:** Target genes of Mother SEC DE miRNAs intersected by four target prediction tools and the 16 target gene interactions generated by compartment analysis in StringDB.

		stringdb::compartment (Confidence score 1-5)									
Gene	Description (Stringdb)	Cytoskeleton	Cytosol	Endoplasmic reticulum	Endosome	Extracellular	Lysosome	Mitochondrion	Nucleus	Plasma membrane	Shared target prediction tools
TEKT3	Tektin 3: Structural component of ciliary and flagellar microtubules. Forms filamentous polymers in the walls of ciliary and flagellar microtubules. Required for progressive sperm mobility (By similarity).	3									miRmapper, miRanda
DDX19A	DEAD-Box Helicase 19A: ATP-dependent RNA helicase involved in mRNA export from the nucleus.								3		miRmapper, miRanda
RAD51D	RAD51 paralog D : Involved in the homologous recombination repair (HRR) pathway of double-stranded DNA	4							4		TargetScan, miRmapper
IL16	Interleukin 16 (IL16), mRNA; Interleukin-16 stimulates a migratory response in CD4+ lymphocytes, monocytes, and eosinophils.					3			3		TargetScan, miRmapper
GLUL	Glutamate-ammonia ligase (GLUL): Essential for proliferation of fetal skin fibroblasts.		4	3				3		4	miRanda, miRWalk
ATP6V1G1	ATPase, H+ transporting, lysosomal 13kDa, V1 subunit G1 (ATP6V1G1)		4				2			4	TargetScan, miRmapper
LHX9	Bos taurus LIM homeobox 9 (LHX9): Involved in gonadal development.								3		TargetScan, miRmapper
UBE2G2	Ubiquitin-conjugating enzyme E2G 2 (UBE2G2): Accepts ubiquitin from the E1 complex and catalyzes its covalent attachment to other proteins.		3	4							miRmapper, miRanda
FKBP1B	FK506 binding protein 1B, 12.6 kDa (FKBP1B): Has the potential to contribute to the immunosuppressive and toxic effects of FK506 and rapamycin.			3							TargetScan, miRmapper
ROBO2	Roundabout, axon guidance receptor, homolog 2										TargetScan, miRmapper
CNP	2',3'-cyclic nucleotide 3' phosphodiesterase (CNP): CNP is the third most abundant protein in central nervous system myelin.					2				2	miRmapper, miRanda
FLOT1	Flotillin 1 (FLOT1): functionally participating in formation of caveolae or caveolae-like vesicles.	2			4				2	3	miRmapper, miRanda
UBE2V1	Ubiquitin-conjugating enzyme E2 variant 1 (UBE2V1)								3		miRmapper, miRanda
SELT	Selenoprotein T: Protects dopaminergic neurons against oxidative stress ans cell death.			4							TargetScan, miRmapper
RNF144B	Ring finger protein 144B (RNF144B): Belongs to the RBR family. RNF144 subfamily.							5			miRmapper, miRWalk
CASR	Parathyroid cell calcium-sensing receptor; G-protein-coupled receptor that senses changes in the extracellular concentration of calcium ions and plays a key role in maintaining calcium homeostasis.									4	miRmapper, miRanda

### 3.4 EV miRNA Profiles: High vs. Low Tick-Resistant (Mother/Sire)

EV samples from high and low tick-resistant mother plasma samples generated 2808 miRNAs. The miRDeep2 identified 350 novel miRNAs from the sequence data, in which more than 70% were shared novel miRNAs between high and low tick-resistant plasma EV samples. Only 44 novel miRNAs expressed in high tick resistant mother plasma EV samples compared to 59 novel miRNAs identified in low tick-resistant mother plasma EV population. The total expressed miRNA list is in **Supplementary file 4** (Abeysinghe, 2021).

The 10 differentially expressed miRNAs from the mother exosomes were included in the list as shown in**Table 4**. The top DE miRNAs from mother exosomes, miR-449a-5p, miR-2285-4-3p and miR-12000-5p are highly expressed in high tick resistant mother EVs than low tick-resistant mother EV population in which the pattern is similar as in **Figure 3A**.

**Figure 4:**
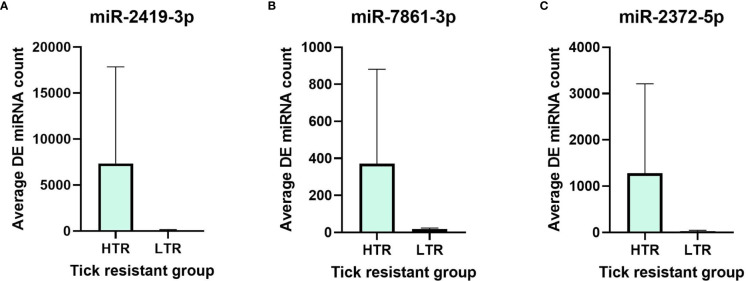
Differential expression of top 3 DE miRNAs **(A)** miR-2419-3p, **(B)** miR-7861-3p and **(C)** miR-2372-5p between high tick resistance (HTR) and low tick resistance (LTR) mother EV samples.

The top three miRNAs which were above the threshold (FDR < 0.05) from EdgeR DGE analysis are shown in **Figure 4** below. The average miRNA count of miR-2419-3p, miR-7861-3p and miR-2372-5p were higher in high tick resistant compared to low tick resistant mother EV population.

### 3.5 EX and Non-EX miRNA Profiles: Offspring of High and Low Tick-Resistant Mother/Sire

**Figure 5:**
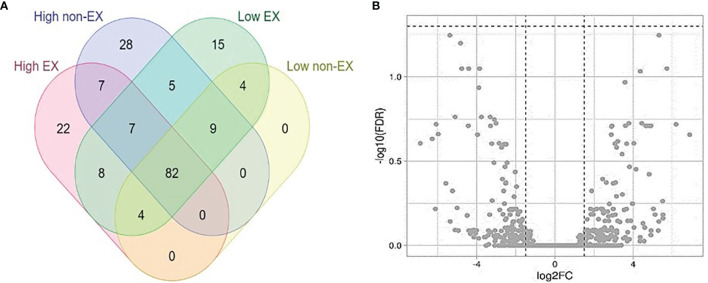
**(A)** Novel miRNAs distributed among high and low tick resistant offspring plasma SEC samples (EX and non-EX). **(B)** Volcano plot of differentially expressed miRNAs in high and low tick resistant offspring SEC samples. The cut off values : [Y axis -log10(FDR) cut off is 1.3 (FDR < 0.05); X axis −1.5 ≤logFC ≥ 1.5].

A total of 196 novel miRNAs were identified in the offspring of high and low-tick resistant mothers/sires (**Figure 5A**). Most miRNAs were common to both groups (**Figure 3A**). Out of 2254 DE miRNAs between offspring of high and low tick-resistant mothers/sires, none were below the FDR cut-off in both EX and non-EX samples (**Figure 5B**). However, the 10 DE miRNAs from mother exosome samples were present in the DE offspring miRNA list. The total list of miRNAs is provided in **Supplementary file 5** (Abeysinghe, 2021).

## 4 Discussion

This is the first study to evaluate miRNA profiles of high and low tick-resistant cattle. Inflammation due to tick infestation may disrupt bovine immune system function and thus facilitate differential expression of miRNAs in low tick-resistant cows. It is interesting to note that differential expression of miRNA is observed at the end stage after tick burden has been established, while DE miRNAs are conserved in the offspring their expression levels are not significantly altered. Further studies may sample cows at an earlier timepoint prior to tick exposure in order to assess any biological vulnerabilities which may point to a predisposition to low tick-resistance.

### 4.1 Differential Expression of miRNA in High and Low Tick-Resistant Cattle

#### 4.1.1 Mir-449a

Mir-449a was highly expressed in maternal high tick-resistant EX. It is commonly associated with cell death, cell-cycle arrest and differentiation, but has also been implicated in many other biological pathways (Lizé et al., 2011). For example, mir-449a acts as a tumor suppressor by inhibiting inflammation and tumor metastasis (Vejnar et al., 2013; Fan et al., 2016). A recent study established that miR-449a is DE in beef cattle with divergent feed efficiency phenotypes (Mukiibi et al., 2020). Finally, in a bovine endometrial receptivity study involving both *in vivo* (IVV) and *in vitro* (IVT) produced embryos, miR-449a was found to be DE based on whether embryos were IVV or IVT derived (Ponsuksili et al., 2014). As such, mir-449a may be considered as a kind of master regulator of a diverse array of biological processes.

#### 4.1.2 Altered Signaling Pathways Are Associated With Inflammation and the Cell Cycle

The 10 DE miRNAs in the current study regulate inflammatory-related pathways and suggest that perturbations to these pathways are related to tick-resistance. For example, the NFķβ signaling pathway and chemokine signaling pathway are both inflammatory pathways that are affected by novel and differentially expressed miRNA in this study. Additionally, the Ras, PI3KT-Akt, and pathways related to cancer are interconnected pathways and known to be related to cell proliferation and differentiation (Downward, 2003; Hemmings and Restuccia, 2012). Therefore, it is possible that low tick resistance is linked to dysregulation of the immune system and pathways related to normal cell cycle function, leading to the inability of the host cattle to reject tick infiltration.

#### 4.1.3 Plasma EV and EX Exhibit Unique Differentially Expressed miRNA Profiles

The 10 DE miRNAs determined in maternal EX samples were also found in maternal EV samples. However, these 10 DE miRNAs were not among the top DE miRNAs in the maternal EV samples, which supports the idea that EX represent a subpopulation of EVs. Larger vesicles, for example microvesicles (diameter >200 nm) may indeed carry more genetic material, including miRNA. Additionally, the 100,000 *rcf* UC pellet may contain circulating miRNA and other RNA fragments. A recent study illustrates the presence of higher number of EX proteins using the UC+SEC isolation and enrichment strategy for blood plasma EX (Turner et al., 2021). UC followed by SEC of plasma samples may therefore contain uniquely sorted exosomal cargo and provides a better cross-section of genetic materials including miRNA. Differential miRNA expression in EX and non-EX samples suggest the involvement of miRNA in the regulation of many different biological functions at both the cellular and systemic level (Zhang et al., 2019; Abeysinghe et al., 2020).

Highly expressed miRNAs from maternal EV populations relates to key bovine traits of interests. An Irish study has revealed polymorphisms in bovine miR-2419 modify its binding properties to target genes related to milk production (Braud, 2017). A recent multi-omics analysis shows that miR-2419-3p is associated with muscle fatty acid traits of Nelore cattle, which may exhibit regulatory function at mRNA or protein level (Cardoso et al., 2021). In a previous study, miR-7861 was differentially expressed as a unique miRNA in Bovine serum compared to EX (Zhao et al., 2016).

#### 4.1.4 Differential Expression of EX miRNA Is Not Heritable

The lack of miRNA differential expression in offspring plasma EX could be related to environmental factors, fewer exposure to ticks or calf immune system immaturity rather than being a genetic trait (Yu et al., 2018). A low correlation of circulating plasma miRNA transcript level between human mother-child duos has been observed in a recent study (Dypås et al., 2020). Future studies may perform sequential sample of the same cattle throughout their lifetime, to provide a time course of the changes occurring at a physiological level that leads to high or low tick-resistance.

This is the first exploratory study on EV and EX vs non-EX miRNA expression profiles of cows and their offspring based on a novel tick scoring system. As a step further, proteomic studies are currently ongoing to ascertain the plasma EX protein expression profiles between the high and low-tick resistant cattle using the same animals used in this study (unpublished data). Further studies expanding the animal number may validate and follow on from the basis of this work to identify the potential role of miRNA as biomarkers or regulators of tick resistance in cattle.

## Data Availability Statement

The datasets presented in this study can be found in online repositories. The names of the repository/repositories and accession number(s) can be found in the article/**Supplementary Material**.

## Ethics Statement

The animal study was reviewed and approved by Animal Welfare Unit, UQ Research and Innovation, the University of Queensland (UQCCR/459/16). Written informed consent was obtained from the owners for the participation of their animals in this study.

## Author Contributions

Conceptualization, PA, NT, HP, KV, NC, NM, MM, and JL. Investigation, PA. Writing—original draft preparation, PA. Writing—review and editing, NT, KV, MM, and JL. Visualization, PA. Supervision, MM and JL. All authors have read and agreed to the published version of the manuscript.

## Funding

This project is funded by Advance Queensland Innovative Partnership (AQIP01115-16RD1) program. Nindooinbah Pastoral Company partnered in this project. PA and NT are supported by a student scholarship from the Australian Research Council (Grant No: ARC LP160101854) and QUT HDR Tuition fee sponsorship. Our laboratory experiments were funded, in part, by funding from a partnership fund (DRCX1302) between the New Zealand Ministry of Business, Innovation and Employment and New Zealand dairy farmers through DairyNZ Inc.

## Conflict of Interest

Authors NC and MM were employed by company Nindooinbah Pastoral Company.

The remaining authors declare that the research was conducted in the absence of any commercial or financial relationships that could be construed as a potential conflict of interest.

## Publisher’s Note

All claims expressed in this article are solely those of the authors and do not necessarily represent those of their affiliated organizations, or those of the publisher, the editors and the reviewers. Any product that may be evaluated in this article, or claim that may be made by its manufacturer, is not guaranteed or endorsed by the publisher.
